# In a Hurry

**Published:** 1876-05

**Authors:** 


### MEDICAL PRINCIPLES.

There are certain general principles in medicine, few and simple, easily understood, and remembered without difficulty; but instead of mastering these, the masses prefer to lumber up their memories with innumerable, and often ridiculous applications of them. We name one at this time.

Poultices are of very extended application ; that of a live chicken cut open and applied instantly, is believed by some to possess extraordinary virtue ; the entrails of a frog are in great esteem by others ; but how a frog is to be obtained in winter, is not explained ; while a live chicken would be rather an expensive application in a city. Scraped potatoes are advised by some as possessing very great "drawing" powers. Dogs get well of their wounds without poultices. Dogs were the doctors who attended Lazarus ; and what is more than can be said of some modern doctors, they treated him as they did themselves, and, no doubt, with advantage. Dogs lick their sores. It is the instinct of Nature. Now let us not run off in the manner of the ignorant and superstitious, and imagine that there is some mysterious virtue in a dog's tongue, some magnetic agency passing back and forth ; but let us look with our eyes open, and in the light of a few well-known facts. Take a common boil, about which most of us have had a feeling experience—the surrounding skin is dry, hot, and hard ; in its natural state, it is moist and soft. To bring it to its natural state again, we have only to remove the dryness, and reduce it to its natural temperature, and what more appropriate than simple soft water, rained or distilled. But to get the full virtue of the application, it should be kept moist all the time ; this would require constant attention, the incessant applying of

